# Successful antipsychotic dose tapering leading to better cognition in patients with remitted psychosis: Results of Guided Antipsychotic Reduction to Reach Minimum Effective Dose (GARMED) trial

**DOI:** 10.1017/S0033291725101591

**Published:** 2025-08-27

**Authors:** Chun-I Liu, Chih-Min Liu, Ming H. Hsieh, Yi-Ting Lin, Yi-Ling Chien, Tzung-Jeng Hwang, Ko Yen, Chen-Chung Liu

**Affiliations:** 1 Department of Psychiatry, National Taiwan University Hospital, Taipei, Taiwan; 2 Department of Psychiatry, College of Medicine, National Taiwan University, Taipei, Taiwan

**Keywords:** antipsychotics, cognitive function, dose–response, remission, tapering

## Abstract

**Background:**

In patients with remitted psychosis, the dosage of antipsychotics can be lowered without increased risk of relapse. Whether dose tapering can lead to improved cognition is unclear. We compared changes in cognitive performance between patients undergoing dose tapering and those receiving a fixed maintenance dose.

**Methods:**

A 2-year prospective trial of patients with stable schizophrenia-related psychotic disorders was conducted: one group received guided dose reduction (GDR) and one group received maintenance treatment. Cognitive function was assessed using the Wechsler Adult Intelligence Scale-Third Edition, Mandarin Chinese version, at baseline, 1, and 2 years. The relations between the ratio of reduced dose and the extent of cognitive improvement were examined by Spearman’s correlation coefficient. We also examined cognitive performance between aripiprazole (ARI) users and non-ARI users.

**Results:**

GDR patients exhibited significantly greater improvements in total intellectual quotient (IQ), particularly working memory, and information and arithmetic subtest scores, with no significant difference in relapse rates between groups. Statistically significant dose–response correlations were found between the degree of dose reduction and improvements in total IQ (*n* = 72, *r* = 0.242, *p* = 0.041), Working Memory Index (*n* = 72, *r* = 0.284, *p* = 0.016), and Arithmetic subtest (*n* = 72, *r* = 0.295, *p* = 0.012). There were no differences in cognitive changes between ARI users and non-users.

**Conclusions:**

Lowering antipsychotic dosage may ameliorate patient performance in several cognitive domains. This finding is worthy of consideration while evaluating the risk-to-benefit ratio of tapering antipsychotics in patients with remitted psychosis.

## Introduction

Antipsychotic medications are a cornerstone of treatment for psychosis, and primarily target dopamine D2 receptors to alleviate symptoms such as delusions, hallucinations, and disorganized thinking. However, chronic use of antipsychotics has been linked to cognitive impairments, especially in the domains of executive function, speed of information processing, memory, and attention (Haddad, Salameh, Sacre, Clément, & Calvet, 2023; Zhang et al., 2024). D2 receptor antagonism plays a critical role in mediating these effects by reducing dopamine signaling in brain regions associated with cognition, such as the prefrontal cortex (Allott, Chopra, Rogers, Dauvermann, & Clark, 2024; Feber et al., 2025).

Different types of antipsychotics are associated with varying degrees of cognitive impairment. This variability may be influenced by factors such as the specific receptor profiles of the medications, their effects on neurotransmitter systems, and the individual patient’s clinical condition (Feber et al., 2025). In recent years, several studies have proposed that newer-generation antipsychotics, such as D2 partial agonists, may have different cognitive effects compared to traditional antipsychotics, but the overall impact of antipsychotic treatment on cognition can differ widely depending on the type and dosage of the medication (García-Fernández et al., 2024; Hori et al., 2006; Husa et al., 2017).

Previous studies have demonstrated a complex relation between the mechanism of D2 receptor occupancy, cognitive dysfunction, and the severity of the psychotic disorder (Gerretsen, Takeuchi, Ozzoude, Graff-Guerrero, & Uchida, 2017). While a D2 antagonist is effective in managing positive symptoms, it has been associated with cognitive impairments and diminished functional outcomes in some patients (Allott et al., 2023; Mehta, Montgomery, Kitamura, & Grasby, 2008; Sakurai et al., 2013). Thus, partial agonists, such as aripiprazole (ARI), may offer a more balanced approach to dopamine modulation, potentially mitigating cognitive decline while reducing the risk of relapse (Elie et al., 2010).

Tapering off antipsychotic medication has garnered attention as a potential strategy to reach functional recovery, especially in patients with remitted psychosis (Koops et al., 2024). Various tapering protocols have been proposed, ranging from gradual dose reductions to complete discontinuation, with the aim of minimizing withdrawal effects and relapse risk (Leucht, Bighelli, Siafis, Schneider-Thoma, & Davis, 2023; O’Neill et al., 2024). Studies examining long-term tapering suggest that tapering may carry an equal risk of relapse compared to maintenance treatment (MT), while simultaneously resulting in improvements in cognitive and functional outcomes (Begemann et al., 2020; Horowitz, Jauhar, Natesan, Murray, & Taylor, 2021). However, the tapering process requires careful monitoring, as abrupt or overly aggressive reductions can lead to relapse or exacerbation of psychotic symptoms (Béchard et al., 2024; McCutcheon et al., 2024). The impact of dose tapering on social cognition, a critical component of functional recovery, has also produced inconclusive results, underscoring the need for further investigation to determine the optimal approach for balancing cognitive improvements with relapse prevention (Moncrieff et al., 2023; Schlier et al., 2023). As a result, a gradual, long-term, and personalized tapering plan tailored to each patient’s clinical stability and overall health is essential (Horowitz & Moncrieff, 2024; Mølgaard, Nielsen, Roed, & Nielsen, 2024).

Several studies have explored the effects of antipsychotic dose reduction on cognitive outcomes, with emerging evidence suggesting that cognitive functions, particularly working memory and executive control, may improve following successful tapering (Omachi & Sumiyoshi, 2018; Zhou, Li, Li, Cui, & Ning, 2018). Cognitive improvements have been associated with the transition from polypharmacy to monotherapy, as well as the reduction of antipsychotic-induced dopamine blockade, allowing for better neural plasticity and brain function recovery (Hori et al., 2013). However, the extent of cognitive improvement appears to be dependent on factors such as the duration of antipsychotic exposure, the tapering protocol used, and individual patient characteristics (Kawai et al., 2006; Singh et al., 2022). Understanding the relation between antipsychotic tapering and cognition may provide critical insights into optimizing treatment strategies for patients in long-term remission from psychosis.

Previously, we conducted a guided dose-tapering trial using an exponential dose reduction protocol, and a substantial proportion of patients with psychosis remained in remission and reported better subjective well-being and functioning (Liu et al., 2023a, 2023b). We also verified the dose reduction using therapeutic drug monitoring, which showed that a lower-than-expected dose, as well as plasma drug concentration, could provide adequate prophylactic effects in a subset of remitted patients receiving ARI (Liu et al., 2024). Additionally, we have measured neurocognitive functioning at baseline and annually, so in this study, we aimed to (1) compare changes of cognitive performance between patients undergoing dose tapering and those receiving a fixed maintenance dose; (2) investigate the relation between the degree of dose reduction and the extent of improved cognitive function; and (3) explore whether there are any specific differences in cognitive outcomes between tapering D2 antagonists and D2 partial agonists.

## Methods

### Study design

This study (NCT03248180) was pragmatically designed as an open-label, randomized, prospective cohort trial that included patients diagnosed with schizophrenia-spectrum disorders. The study protocol, briefly described below, has been published elsewhere (Liu et al., 2022). Participants were allocated randomly in a 2:1 ratio to either the guided dose reduction (GDR) group or the MT group (MT1). Additionally, individuals who met the inclusion criteria but chose not to partake in dose reduction were invited to join a naturalistic comparison group (MT2), where they continued on their existing antipsychotic regimen. Participant recruitment was conducted at National Taiwan University Hospital, Taipei, Taiwan, from August 2017 to September 2022.

### Participants

The study population consisted of patients diagnosed with schizophrenia-spectrum disorders based on Diagnostic and Statistical Manual of Mental Disorders-Fifth Edition (DSM-5) criteria. Eligible participants were in a remitted state of psychosis, receiving a fixed dose of antipsychotic medication for at least 3 months before recruitment. The remitted state was defined as patients without unstable symptoms (a score of ⩾4 on any of the 5 Positive and Negative Syndrome Scale [PANSS] items – P1: delusion, P2: conceptual disorganization, P3: hallucination, G9: unusual thought, G5: mannerism and posturing, or a score of ⩾5 on any other PANSS items) were excluded. Other exclusion criteria were as follows: (1) non-remitted patients, admission to an acute psychiatric ward within the past 6 months; (2) concurrent use of mood stabilizers; (3) a revised dose of psychotropic agents within the previous 3 months; (4) an intellectual quotient (IQ) below 70 before schizophrenia diagnosis; (5) a history of other mental health disorders or substance dependence within the past 6 months; and (6) pregnant or breastfeeding.

Before enrollment, all participants were fully informed of the potential risks and benefits associated with long-term antipsychotic treatment and the rationale for guided dose tapering. Written informed consent was obtained from each participant. The study adhered to the ethical principles outlined in the Declaration of Helsinki and the International Conference on Harmonization Good Clinical Practice guidelines. The protocol, including any amendments, was reviewed and approved by the Research Ethics Committee of National Taiwan University Hospital (REC: 201703002RIND).

### Exponential dose reduction protocol

Participants in the GDR group underwent a gradual tapering of antipsychotics, starting with a 25% reduction from their baseline dosage. Monthly monitoring of psychotic symptoms was performed for 3 months, followed by another 3-month period during which no further dose adjustments were made, totaling 6 months of stabilization. After this period, the current dose was again reduced by 25%, resulting in a total dose reduction to 56.25% (0.75 × 0.75) of the baseline dose. At each time point eligible for the next dose reduction, participants who were in stable condition without psychosis relapse could discuss with their psychiatrists whether to continue reducing the dosage or to maintain the current level through shared decision-making. This cycle of dose reduction was repeated at a minimum of 6-month intervals over the course of the 2-year follow-up. The rationale and further details regarding this dose tapering algorithm were addressed in prior publications (Liu et al., 2022; Liu & Takeuchi, 2020).

### Outcome assessments

For all participants, several subtests from the Mandarin Chinese language version of Wechsler Adult Intelligence Scale, Third Edition (WAIS-III) (Chen, Hua, Zhu, & Chen, 2008), including Information, Arithmetic, Similarities, Block Design, Digit Symbol, and Digit Span, were administered at baseline, at the 1-year follow-up, and at the end of the study (2-year follow-up). These assessments were conducted by three well-trained research assistants who were blinded to the participants’ clinical conditions and previous test results. The raw scores were transformed to standardized scores based on the norm developed in a previous study (Chen et al., 2008). The Working Memory Index was defined as the combined scores of the Arithmetic and Digit Span subtests. We calculated the sum of all the subtest scores, except Digit Span, then transformed it to a standardized score according to the norms provided by Chen et al.’s report to be the estimated total IQ. The total IQ was designated as the primary outcome, while the IQ subtests were considered secondary outcomes. Psychopathological evaluations were carried out using the Mandarin version of the PANSS scale (Cheng et al., 1996), the Clinical Global Impression of Severity (CGI-S), and the Personal and Social Performance (PSP) scale (Wu et al., 2013). These assessments were administered by a trained psychiatrist at the start of the study and again at the end of the 2-year follow-up period.

Participant demographic and clinical information, including diagnoses, age at onset of illness, illness duration, history of admissions, history of relapses, and employment status, were collected at baseline and at the conclusion of the study by extracting data from medical records supplemented with individual interviews conducted by a research assistant. Antipsychotic dosage was documented at each time point of dose adjustment and was compiled annually.

### Statistical analyses

To assess potential bias in group assignment, baseline psychopathology and cognitive function were compared between the two groups using the PANSS, PSP, CGI, and the WAIS-III subtests. Categorical variables were analyzed using the *χ* ^2^- test, while continuous variables were evaluated with analysis of variance. To determine the impact of dose reduction on cognitive function between the GDR and MT groups, analysis of covariance (ANCOVA) was used to control for the possible confounding factors and compare the cognitive changes between groups. Cognitive change was defined as the difference in standardized scores between the last observation and that at baseline. Logistic regression was applied to compare the between-group difference at the end of the study of categorical outcomes. The intent-to-treat analysis was conducted between the GDR and the MT group. Effect size was calculated by using Hedge’s *g.*

Of note, participants in the GDR group were guided to adjust their dosage based on their clinical condition; thus, some patients may have no dose reduction by the end of follow-up. Participants in the MT group may have self-reduced their dosage, resulting in some patients taking a dosage lower than that at their baseline. Thus, we further reclassified the participants into two groups: the actual dose reduction group and the actual fixed-dose maintenance group. Cognitive changes were then compared between these two groups as per protocol analysis.

Spearman’s rank correlation analysis was used to assess the dose–response relation between the ratio of dose reduction and improvements in cognitive subtests. Simple linear regression models were used to predict cognitive improvement based on the degree of dosage reduction. To determine if the impact of dose reduction on cognitive function was different between dopamine D2 partial agonists and other dopamine antagonists, further analyses were performed by comparing the correlation coefficients between dosage reduction and cognitive improvement in the ARI group versus the non-ARI group. The interaction *p*-values were calculated to assess if the effects of dose reduction differed between these two subgroups.

## Results

### Demographic and clinical characteristics

Of the 196 patients assessed for eligibility, 98 patients participated in the study, and 91 of the 98 with complete cognitive assessments were included in the analysis. There were 51 patients in the GDR group, 19 in the MT1 group, and 21 in the MT2 group. Patients in the MT1 and MT2 groups were combined into a single MT group.

**Figure 1.**
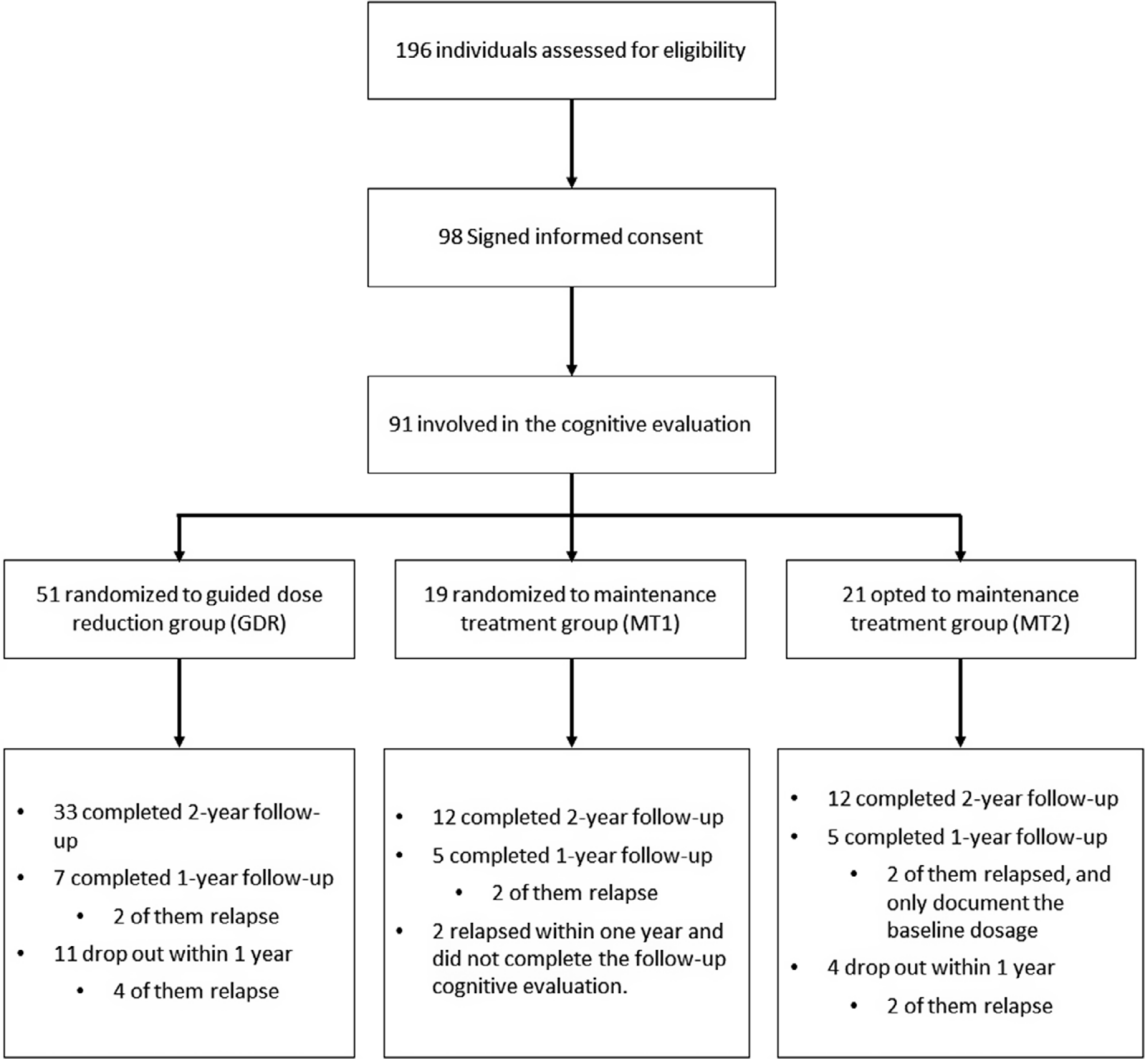
Flow diagram.

Among the 91 participants, 57 completed the 2-year follow-up period. For these participants, the dose reduction ratio was defined as the difference between the dosage at the end of the study and the baseline dosage. Fifteen participants had both baseline and 1-year data of dosage and cognitive assessments, and for these patients, the dose reduction ratio was calculated as the dosage at 1 year minus the baseline dosage. Two participants underwent cognitive assessments at 1 year but had only baseline dosage data available, and thus, they were only included in the comparison between the GDR and MT groups, but not in the analysis of the relation between dosage reduction and cognitive change. Seventeen participants were lost to follow-up in the first year and were excluded from further analysis. The CONSORT diagram of patient participation and inclusion is shown in Figure 1.

The baseline characteristics of the 91 participants are summarized in Table 1. There were no significant differences in antipsychotic dosage, psychopathology, or baseline cognitive function between the GDR and MT groups. The age is older and the duration of illness (DOI) is longer in the MT group. Age and DOI were centered first to avoid multicollinearity, and then treated as confounding factors in the ANCOVA and logistic regression. The antipsychotic medications taken by the participants included fluphenazine, haloperidol, flupentixol, sulpiride, amisulpride, olanzapine, clozapine, risperidone, and paliperidone. The only dopamine D2 partial agonist used was ARI.

**Table 1.** Patient baseline characteristics and cognitive outcomes after a 2-year follow-up

	Maintenance treatment group *n* = 40	Guided dose reduction group *n* = 51	Total *N* = 91	*P*-value
Age, years	38.7 ± 10.70	34.3 ± 9.6	36.2 ± 10.3	0.041^a^
Sex, male	26 (65)	25 (49.0)	51 (56.0)	0.130
Onset age	25.5 ± 9.6	24.7 ± 6.4	25.1 ± 7.9	0.646
History of admission	22 (55)	29 (56.9)	51 (56.0)	0.861
History of relapse	27 (67.5)	34 (66.7)	61 (67.0)	0.934
Duration of illness, years	13.2 ± 8.1	9.6 ± 8.5	11.2 ± 8.5	0.046^a^
Aripiprazole user	16 (40)	15 (29.4)	31 (34.1)	0.295
Employment				0.507
Full-time	22	21	43	
Part-time	7	17	24	
No	11	13	24	
Diagnosis				0.740
Schizophrenia	31	41	72	
Other	9	10	19	
PANSS score	41.4 ± 7.3	40.5 ± 9.7	40.9 ± 8.7	0.648
PSP score	81.3 ± 6.9	80.1 ± 8.4	80.6 ± 7.7	0.464
CGI-S score	1.85 ± 0.74	2.04 ± 0.80	1.96 ± 0.77	0.249
Chlorpromazine ED, mg/d	195 ± 131.2	206.9 ± 133.7	201.7 ± 132	0.672
Total IQ score	96.1 ± 13.7	96.1 ± 14.1	96.1 ± 13.8	0.953
Information subscore	9.87 ± 2.54	9.51 ± 3.28	9.67 ± 2.96	0.399
Arithmetic subscore	8.77 ± 2.71	8.66 ± 2.67	8.71 ± 2.67	0.853
Similarity subscore	9.64 ± 2.07	9.68 ± 2.25	9.66 ± 2.16	0.992
Block Design subscore	10.3 ± 3.3	10.2 ± 3.9	10.3 ± 3.6	0.905
Digit Symbol subscore	8.67 ± 3.27	9.17 ± 3.26	8.94 ± 3.26	0.410
Digit Span subscore	11.2 ± 3.0	11.3 ± 3.6	11.3 ± 3.3	0.791
Working Memory score	20.0 ± 4.9	20.0 ± 5.5	20.0 ± 5.2	0.994

After a 2-year follow-up	Maintenance treatment group *n* = 34	Guided dose reduction group *n* = 40	Total *N* = 74	*P*-value	Hedge’s *g*
Chlorpromazine ED mg /d	178 ± 122.5	124.2 ± 76.3	148.9 ± 103.1	0.022^a^	
Dose reduction ratio	8.5 ± 0.15	38.8 ± 0.22	25.3 ± 0.24	<.001^a^	
Aripiprazole user	12 (35.3)	8 (20)	20 (27)	0.773	
Employment					
Full-time	22	17	39		
Part-time	4	11	15		
No	10	12	22		
Relapse	4 (11.8)	2 (5.00)	6 (8.1)	0.333	
ΔTotal IQ	2.25 ± 7.44	6.28 ± 6.12	4.43 ± 7.01	0.013^a^	0.597
ΔInformation	0.32 ± 1.20	1.05 ± 1.47	0.72 ± 1.39	0.0252^a^	0.538
ΔArithmetic	0.00 ± 1.91	1.03 ± 2.01	0.55 ± 2.01	0.0293^a^	0.523
ΔSimilarity	0.65 ± 1.74	1.15 ± 1.90	0.92 ± 1.83	0.236	0.275
ΔBlock Design	0.21 ± 2.06	0.85 ± 1.66	0.55 ± 1.87	0.139	0.348
ΔDigit Symbol	0.76 ± 2.03	0.90 ± 2.18	0.84 ± 2.10	0.784	0.064
ΔDigit Span	0.53 ± 1.38	0.85 ± 2.63	0.70 ± 2.14	0.517	0.149
ΔWorking Memory	0.5 ± 2.55	1.88 ± 2.99	1.24 ± 2.86	0.040^a^	0.492

*Note:* Data are reported as mean ± standard deviation, or count (percentage).

Cognitive improvements were measured as changes in standard scores between baseline and the last observation.

The *P*-value was calculated after control of age and duration of illness.

Abbreviations: CGI-S, Clinical Global Impression of Symptom Severity; ED, equivalent dose; IQ, intellectual quotient; PANSS, Positive and Negative Syndrome Scale; PSP, Personal and Social Performance Scale.

a Denotes parameters that reached statistical significance within the 95% confidence interval.

### Cognitive changes between the GDR and the MT group

After the end of the 2-year follow-up, the GDR group (*n* = 40) achieved a mean reduction of their baseline antipsychotic dosage of 38.8%, while the MT group (*n* = 34) had a mean reduction of 8.5%. Both groups showed mild improvement in most cognitive subtests. Compared to the MT group, the GDR group exhibited significantly greater cognitive improvements in Total IQ, specifically in the Working Memory Index, as well as Information and Arithmetic subtests, with moderate effect sizes (Table 1). Additionally, there was no significant difference in relapse rate between the two groups.

### Cognitive changes between actual-dose reduction and fixed-dose maintenance groups

When patients were recategorized as actual dose-reduction group (*n* = 42) and fixed-dose maintenance group (*n* = 30), the actual dose-reduction group had statistically significant improvements in Total IQ and the Arithmetic subtest; however, improvements of the Working Memory Index or the Information subtest were not significantly different (Table 2).

**Table 2.** Comparison between the actual dose reduction group and the actual dose maintenance group

	Fixed-dose maintenance *n* = 30	Actual dose reduction *n* = 42	Total *n* = 72	*p*-value	Hedge’s *g*	Correlation coefficient (*r*) with a dose reduction ratio	*p*-value
Chlorpromazine ED mg /d	184.3 ± 129.9	124.8 ± 73.5	149.6 ± 104.4	0.014^a^			
Dose reduction ratio	0 ± 0	43.4 ± 0.2	25.3 ± 0.2	<.001^a^			
Aripiprazole user	9 (30)	10 (23.8)	19 (26.4)	0.307			
Relapse	3 (10)	1 (2.4)	4 (5.6)	0.785			
ΔTotal IQ	2.13 ± 7.47	6.25 ± 6.30	4.53 ± 7.06	0.014^a^	0.605	0.242	0.041^a^
ΔInformation	0.47 ± 1.38	0.95 ± 1.38	0.75 ± 1.39	0.149	0.352	0.221	0.062
ΔArithmetic	−0.30 ± 1.82	1.14 ± 1.95	0.54 ± 2.01	0.002^a^	0.761	0.295	0.012^a^
ΔSimilarity	0.83 ± 1.88	0.95 ± 1.83	0.90 ± 1.84	0.786	0.064	0.076	0.526
ΔBlock Design	0.27 ± 1.96	0.81 ± 1.78	0.58 ± 1.87	0.225	0.292	0.145	0.224
ΔDigit Symbol	0.77 ± 2.08	0.95 ± 2.09	0.88 ± 2.08	0.709	0.089	0.044	0.713
ΔDigit Span	0.73 ± 1.93	0.69 ± 2.34	0.71 ± 2.17	0.934	−0.020	0.060	0.617
ΔWorking Memory	0.47 ± 8.97	1.79 ± 2.58	1.24 ± 2.87	0.055	0.469	0.284	0.016^a^

Data are reported as mean ± standard deviation, or count (percentage).

The *p*-value of between-group difference was calculated after control of age and duration of illness.

Cognitive improvements were measured as changes in standard scores between baseline and the last observation.

Abbreviations: ARI, aripiprazole; ED, equivalent dose; IQ, intellectual quotient.

a Denotes parameters that reached statistical significance within the 95% confidence interval.

### Relation between the degree of reduced dose and the extent of improved cognition

**Figure 2.**
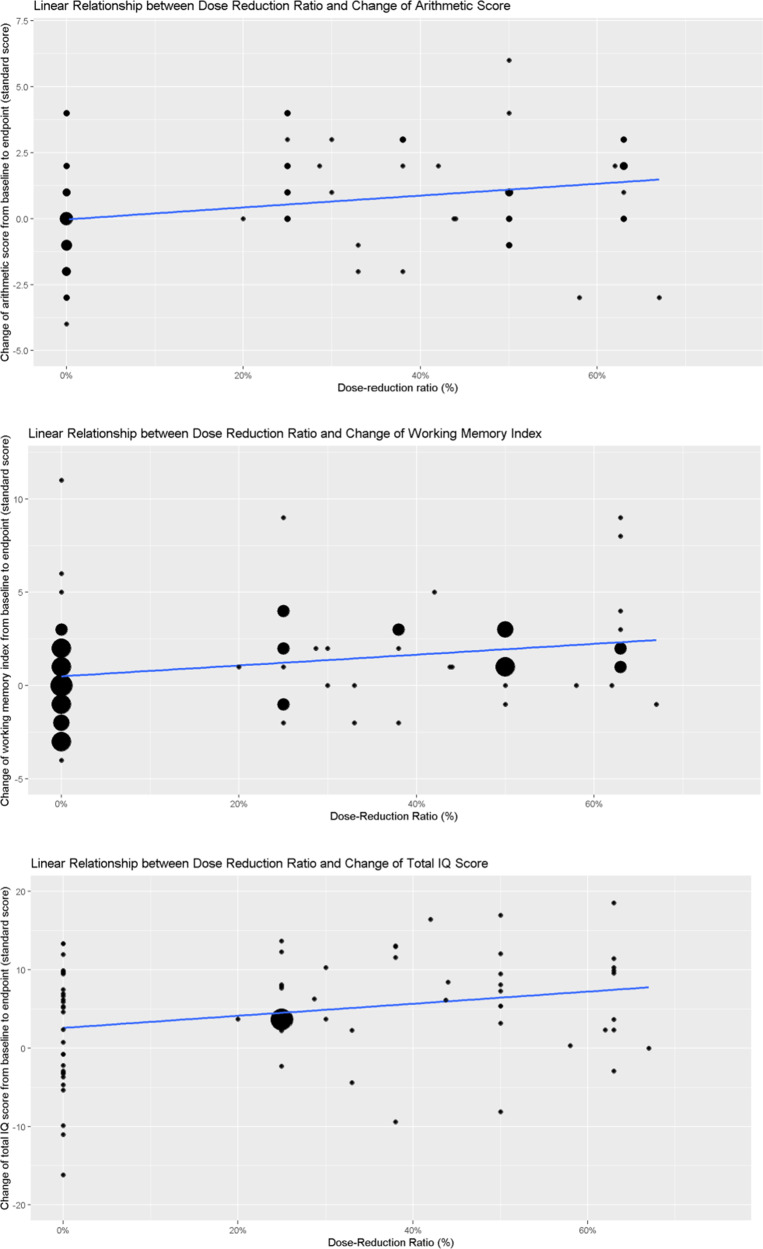
Correlation between cognitive improvement and the dose reduction ratio. The size of the dots reflects overlapping data points.

Spearman’s correlation analyses between the degree of reduced dose and the extent of improved cognitive performance revealed statistically significant results in Total IQ (*n* = 72, *r* = 0.242, *p* = 0.041), Working Memory Index (*n* = 72, *r* = 0.284, *p* = 0.016), and Arithmetic subtest (*n* = 72, *r* = 0.295, *p* = 0.012) (Table 2). A linear regression model was subsequently established for these three domains, and the regression lines are presented in Figure 2.

### Comparison between the ARI and non-ARI groups

**Figure 3.**
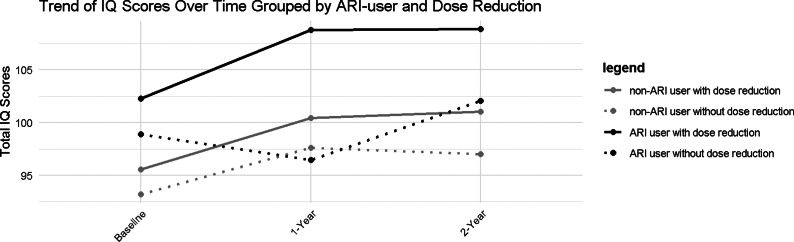
Trajectories of the Total IQ change grouped by ARI user and dose reduction. *Note:* ARI, aripiprazole; IQ, intellectual quotient.

The baseline characteristics between ARI users and non-ARI users were comparable, except that ARI users were younger. ARI users performed better in most cognitive subtests, although differences between the groups were not statistically significant. The differences in changes of cognitive performance between the groups are presented in Supplementary Table 1, including *p*-values for interaction terms (reduction ratio × ARI use) in the correlational analysis. Only significantly improved Digit Symbol subtest scores were seen in non-ARI users, but not in ARI users. The different trajectories of the Total IQ changes among groups categorized as ARI use/non-ARI use are illustrated in Figure 3 and Supplementary Figure 1. The results suggest that the ARI dose reduction group had better performance across all time points, although the differences were not statistically significant. The dose–response relations between dose-reduction ratios and cognitive improvement were similar between the ARI users and non-ARI users (Supplementary Figure 2).

## Discussion

In addition to findings from our previous reports, which provided evidence of improved well-being with no increased risk of relapse under a slow exponential dose tapering algorithm (Liu et al., 2023a, 2023b), with results verified by therapeutic drug monitoring (Liu et al., 2024), this study further shows the impact of dose reduction on cognitive function. Echoing the call for more evidence to understand the mechanisms of antipsychotic-associated cognitive impairment in patients with remitted psychosis (Allott et al., 2024), our results demonstrated that during the remitted phase, lowering antipsychotic dosage may ameliorate cognitive performance in several cognitive domains. There are some important points that deserve discussion.

First, a direct comparison between the GDR group and the MT group revealed a significant difference in cognitive improvement across several domains. Cognitive impairment is a core symptom of schizophrenia, often persists throughout the course of the illness, and is correlated with functional impairment (Kubota et al., 2015; Thomas et al., 2021). Yet, whether the use of antipsychotics can improve or worsen cognitive symptoms remains unresolved. Some studies suggest that high doses are associated with reduced brain volume, while lower doses may enhance cognitive performance and overall functioning (Hulkko et al., 2017; Veijola et al., 2014). Conversely, other studies emphasize that each relapse episode may worsen cognition, supporting the need for high-dose antipsychotic maintenance to prevent relapse (Chan et al., 2022; Cuesta et al., 2022). Our findings demonstrated that the dose reduction group exhibited significantly greater cognitive improvement compared to the maintenance group (Table 1). This result is still present when comparing the actual dose reduction group and the actual fixed-dose maintenance group (Table 2), suggesting that gradual tapering of antipsychotics may be a feasible approach to recover cognitive functioning for patients with remitted psychosis.

Second, the positive outcomes following the dose reduction strategy were also associated with the extent of reduced dosage, exemplified by positive dose–response relations between the ratio of reduced dose and the extent of improved cognitive performance (Figure 2). Previous studies have found that cumulative lifetime antipsychotic dose correlates with cognitive decline, while the actual effect may be confounded by the disease severity and duration (Hulkko et al., 2017; Husa et al., 2014). Neuroimaging studies have also shown that a lower cumulative antipsychotic dose is associated with larger white matter volume, while the illness severity should also be taken into account to clarify the definitive effect of cumulative antipsychotic dosage (Emsley et al., 2023; Huhtaniska et al., 2017). Our findings from a 2-year trial support the impact of lower antipsychotic dose with better cognition, at least in a group of stable patients with remitted psychosis. More notably, our results suggest a chance to ameliorate the negative impact of antipsychotics on cognitive function by dose tapering. Nonetheless, how to reach an optimal extent of dose reduction to maximize cognitive benefits with minimized risk of relapse remains a big challenge.

Third, this is the first study to examine the effects of dose reduction specifically for dopamine D2 receptor partial agonists. Previous literature suggests that cognitive impairment is associated with the effects of D2 receptor blockade, while D2 partial agonists may have the potential to improve cognitive function due to their modulating effects on dopamine activity (Bliźniewska-Kowalska & Gałecki, 2024; Sakurai et al., 2013). However, prior studies have not investigated whether dose reduction effects differ between D2 partial agonists and D2 antagonists. In our subgroup analysis, a different impact on cognition was observed only in the Digit Symbol subtest between the D2 antagonist and D2 partial agonist groups (Supplementary Table 1). Notably, at baseline, the ARI-treated patients already had better cognitive performance at doses equivalent to their non-ARI counterparts, and continued to improve during dose reduction, while the non-ARI users exhibited improved cognitive performance after dose reduction to a level comparable to that of the non-tapering ARI users (Figure 3). Since the *p*-values for interaction terms are nonsignificant in Total IQ and all subtests, it implies that dose tapering may be beneficial to patients treated with either D2 partial agonists or D2 antagonists.

There are several limitations to this study. First, the patients were examined during the remitted phases for dose reduction; thus, the findings do not apply to patients in an acute phase of psychosis who were not considered candidates for dose reduction. Second, the baseline doses of our participants were relatively low, so the impact on cognitive functioning might not be as evident as studies in patients treated with a higher antipsychotic dose. Also, the potential differences in impact following dose tapering between D2 partial agonists and D2 antagonists might be obscured due to relatively low starting doses. Third, this study focused on cognitive domains that have been well-documented in patients with schizophrenia; other cognitive functioning, such as social cognition, verbal learning, and memory, which are also well-correlated with patient functioning, was not fully explored. Lastly, the co-administration of psychotropic agents such as benzodiazepines or anticholinergics was not well-controlled, and their use could have influenced the outcomes. The aforementioned limitations require long-term follow-up to confirm how durable such improvement can be sustained, and whether they can be translated to better personal, social, and occupational functioning.

In summary, the findings of this study suggest that a cautious exponential dose reduction strategy can lead to improved cognition as demonstrated by significant differences in outcomes between patients undergoing dose tapering and those receiving fixed-dose maintenance, as well as an evident dose–response relation between the reduced dosage and improved cognitive performance. Patients treated with either D2 partial agonists or D2 antagonists could gain benefits via this dose-tapering approach at a relatively low dose range. Whether patients receiving higher-dose D2 antagonists can improve more compared to those receiving D2 partial agonists after dose tapering warrants further investigation.

## Supporting information

Liu et al. supplementary material Liu et al. supplementary material

## Supplementary material

The supplementary material for this article can be found at http://doi.org/10.1017/S0033291725101591.
