# Supplementary material for: Successful antipsychotic dose tapering leading to better cognition in patients with remitted psychosis: Results of Guided Antipsychotic Reduction to Reach Minimum Effective Dose (GARMED) trial
Source: Psychol Med. 2025 Aug 27;55:e247. doi: 10.1017/S0033291725101591 (PMC12404327; doi:10.1017/S0033291725101591)
Supplement: Liu et al. supplementary material [file S0033291725101591sup001.docx]

**Supplement Table 1. Comparison between ARI users and non-ARI users**

|  | Non-ARI user  n = 53 | ARI user  n = 19 | Total  n = 72 | p value | p value for interaction  (Reduction ratio x ARI use) |
| --- | --- | --- | --- | --- | --- |
| Age, years | 39 ± 10.9 | 33.3 ± 7.1 | 37.5 ± 10.3 | 0.037* |  |
| Sex, Male | 27 (51) | 14 (73.7) | 41 (56.9) | 0.088 |  |
| Onset age | 26.1 ± 8.9 | 24.1 ± 6.1 | 25. 6 ± 8.3 | 0.359 |  |
| History of admission | 35 (66) | 9 (47.7) | 44 (61.1) | 0.156 |  |
| History of relapse | 36 (67.9) | 14 (73.7) | 50 (69.4) | 0.646 |  |
| Duration of illness, years | 12.9 ± 9.2 | 9.2 ± 5.5 | 11.9 ± 8.5 | 0.104 |  |
| Dose Reduction Ratio | 32 (60.4) | 10 (52.6) | 42 (58.3) | 0.251 |  |
| Employment |  |  |  | 0.051 |  |
| Full-time | 13 | 6 | 19 |  |  |
| Part-time | 24 | 12 | 36 |  |  |
| No | 16 | 1 | 17 |  |  |
| Diagnosis |  |  |  | 0.385 |  |
| Schizophrenia | 44 (83) | 14 (73.7) | 58 (80.6) |  |  |
| Other | 9 (17) | 5 (26.3) | 14 (19.4) |  |  |
| Relapse | 3 (5.7) | 1 (5.3) | 4 (5.6) | 0.949 |  |
| PANSS score at T0 | 39.8 ± 6.4 | 40.5 ± 7.9 | 39.9 ± 6.8 | 0.695 |  |
| PSP score at T0 | 80.4 ± 6.9 | 83.8 ± 6.7 | 81.3 ± 6.9 | 0.060 |  |
| CGI-S score at T0 | 2 ± 0.76 | 1.68 ± 0.67 | 1.92 ± 0.75 | 0.114 |  |
| Chlorpromazine ED mg/d at T0 | 205.4 ± 141.6 | 204.1 ± 103.6 | 205.1 ± 131.9 | 0.971 |  |
| Total IQ score at T0 | 94.7 ± 13 | 100.7 ±13.8 | 96.3 ± 13.4 | 0.093 |  |
| Information subscore at T0 | 9.90 ± 3.03 | 9.36 ± 2.11 | 9.76 ± 2.81 | 0.481 |  |
| Arithmetic subscore at T0 | 8.58 ± 2.80 | 9.0 ± 2.58 | 8.69 ± 2.73 | 0.567 |  |
| Similarity subscore at T0 | 9.48 ± 2.16 | 10.3 ± 2.1 | 9.69 ± 2.15 | 0.176 |  |
| Block Design subscore at T0 | 9.90 ± 3.35 | 11.5 ± 3.7 | 10.3 ± 3.5 | 0.084 |  |
| Digit Symbol subscore at T0 | 8.54 ± 2.87 | 10.1 ± 3.6 | 8.94 ± 3.14 | 0.072 |  |
| Digit Span subscore at T0 | 10.7 ± 3.2 | 11.8 ± 3.6 | 11.0 ± 3.3 | 0.232 |  |
| Working Memory score at T0 | 19.3 ± 5.1 | 20.8 ± 5.5 | 19.7 ± 5.2 | 0.290 |  |
| ΔTotal IQ | 5.17 ± 6.86 | 2.75 ± 7.51 | 4.53 ± 7.06 | 0.204 | 0.228 |
| ΔInformation | 0.74 ± 1.52 | 0.79 ± 0.98 | 0.75 ± 1.39 | 0.887 | 0.810 |
| ΔArithmetic | 0.36 ± 1.94 | 1.05 ± 2.17 | 0.54 ± 2.01 | 0.199 | 0.757 |
| ΔSimilarity | 0.94 ± 1.94 | 0.79 ± 1.58 | 0.90 ± 1.84 | 0.757 | 0.481 |
| ΔBlock Design | 0.74 ± 1.77 | 0.16 ± 2.12 | 0.58± 1.87 | 0.250 | 0.587 |
| ΔDigit Symbol | 1.26 ± 1.96 | -0.21 ± 2.04 | 0.88 ± 2.08 | 0.007* | 0.191 |
| ΔDigit Span | 0.66 ± 2.08 | 0.84 ± 2.43 | 0.81 ± 2.17 | 0.756 | 0.062 |
| ΔWorking Memory | 1.04 ± 2.63 | 1.79 ± 3.47 | 1.24 ± 2.87 | 0.331 | 0.424 |

Data are reported as mean ± standard deviation, or count (percentage).

ARI, aripiprazole; CGI-S, clinical global impression of symptom severity; ED, equivalent dose; IQ, intellectual quotient; PANSS, Positive and Negative Syndrome Scale; PSP, Personal and Social Performance Scale; T0, baseline.

*Denotes parameters that reached statistical significance within the 95% confidence interval.


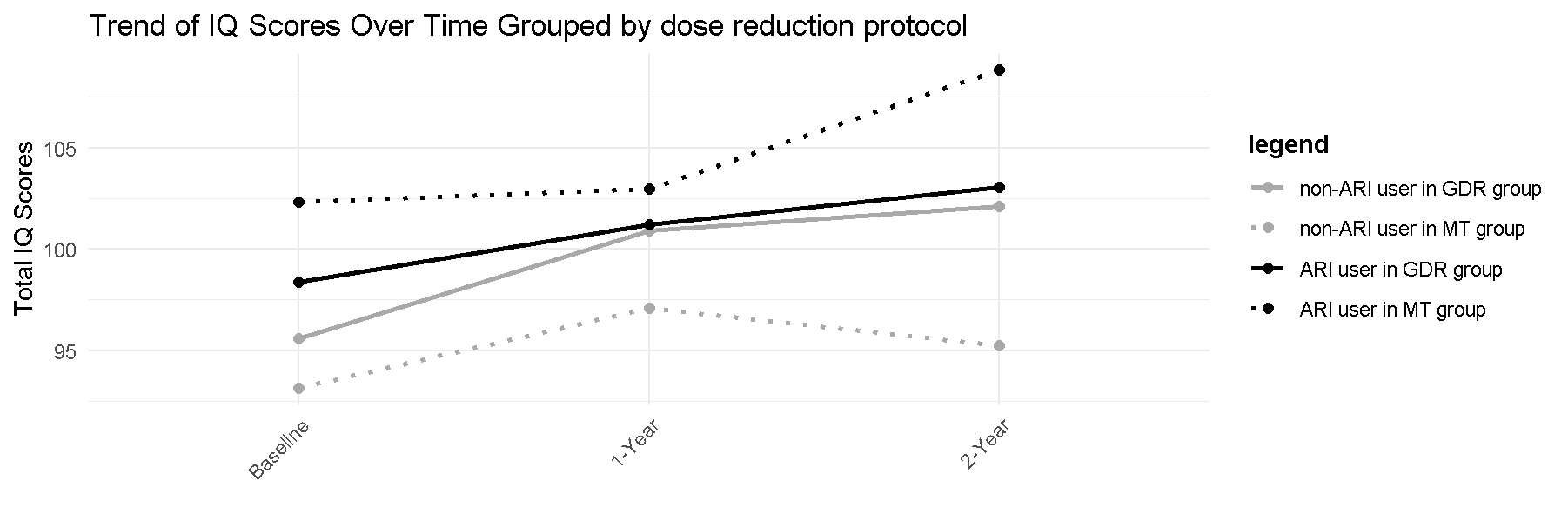


Supplement Figure 1. Trajectories of the Total IQ change of grouped by dose reduction protocol


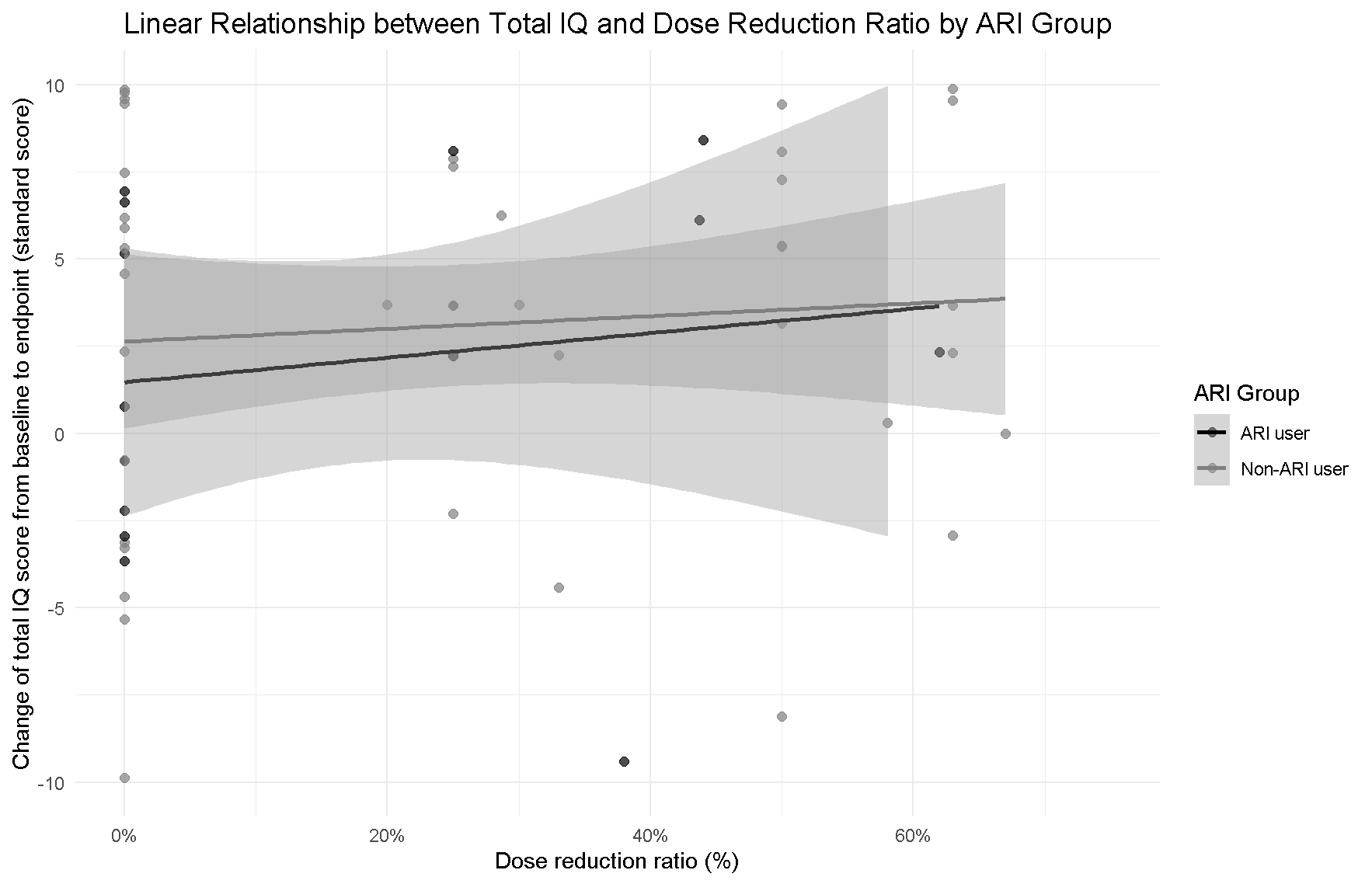


Supplement Figure 2. Correlations between Total IQ change and Dose Reduction Ratio

ARI, aripiprazole. IQ, intellectual quotient.
